# *Blastocystosis* in patients with gastrointestinal symptoms: a case–control study

**DOI:** 10.1186/1471-230X-12-122

**Published:** 2012-09-10

**Authors:** Ayhan Hilmi Cekin, Yesim Cekin, Yesim Adakan, Ezel Tasdemir, Fatma Gulsun Koclar, Basak Oguz Yolcular

**Affiliations:** 1 Deparment of Gastroenterology, Antalya Training and Research Hospital, Antalya, Turkey; 2 Department of Microbiology, Antalya Training and Research Hospital, Antalya, Turkey; 3 Department of Internal Medicine, Antalya Training and Research Hospital, Antalya, Turkey; 4 Department of Parositology, Antalya Training and Research Hospital, Antalya, Turkey; 5 Department of Biostatistics and Medical Informatics, Akdeniz University Faculty of Medicine, Antalya, Turkey

**Keywords:** Ulcerative colitis, Crohn’s disease, Irritable bowel syndrome, Blastocystis spp

## Abstract

**Background:**

*Blastocystosis* is a frequent bowel disease. We planned to to evaluate the prevalence of *Blastocystis spp.* in patients who applied to the same internal medicine-gastroenterology clinic with or without gastrointestinal complaints to reveal the association of this parasite with diagnosed IBS and IBD.

**Methods:**

A total of 2334 patients with gastrointestinal symptoms composed the study group, which included 335 patients with diagnosed inflammatory bowel disease and 877 with irritable bowel syndrome. Patients without any gastrointestinal symptoms or disease (n = 192) composed the control group. Parasite presence was investigated by applying native-Lugol and formol ethyl acetate concentration to stool specimens, and trichrome staining method in suspicious cases.

**Results:**

*Blastocystis spp.* was detected in 134 patients (5.74%) in the study group and 6 (3.12%) in the control group (p = 0.128). In the study group, *Blastocystis spp.* was detected at frequencies of 8.7% in ulcerative colitis (24/276), 6.78% in Crohn’s disease (4/59), 5.82% in irritable bowel syndrome (51/877), and 4.9% in the remaining patients with gastrointestinal symptoms (55/1122). *Blastocystis spp.* was detected at a statistically significant ratio in the inflammatory bowel disease (odds ratio [OR] = 2.824; 95% confidence interval [CI]: 1.149-6.944; p = 0.019) and ulcerative colitis (OR = 2.952; 95% CI: 1.183-7.367; p = 0.016) patients within this group compared to controls. There were no statistically significant differences between the control group and Crohn’s disease or irritable bowel syndrome patients in terms *Blastocystis spp.* frequency (p = 0.251, p = 0.133).

**Conclusions:**

*Blastocystosis* was more frequent in patients with inflammatory bowel disease, especially those with ulcerative colitis. Although symptomatic irritable bowel syndrome and Crohn’s disease patients had higher rates of *Blastocystis spp.* infection, the differences were not significant when compared to controls.

## Background

*Blastocystis spp.* is a unicellular protozoan found in the large intestine in humans. While Blastocystis infection is common worldwide, it is observed more frequently in tropical climates and developing countries with a wide host population including mammals, birds, reptiles, and arthropods [1]. The parasite spreads through the fecal-oral route particularly under poor hygiene conditions [2] and use of unboiled water has been considered to have a significant role in the spread of the infection [3]. Six forms have been defined to date in the culture and direct stool analysis while the vacuolar form is the most frequently form encountered in the stool [4]. Diagnosis of infection is generally made by demonstrating the vacuolar form in the direct microscopic examination of the stool with presence of more than five parasites in a microscope field at X40 magnification accepted as pathogenic [5]. Until recently, it was accepted that only one *Blastocystis* species (*Blastocystis hominis*) infected human beings. However, in recent years, it was demonstrated that nine different species may infect humans [6].

Untreated *Blastocystis spp.* can accommodate in the human gastrointestinal system for a few weeks up to a few years [2]. The pathogenic potential of *Blastocystis spp.* is still a debated issue due to the fact it is the most frequently encountered protozoan in healthy individuals as well as patients with gastrointestinal symptoms [1,4]. *Blastocystis spp.* infection is generally asymptomatic. Symptomatic cases are characterized by nonspecific gastrointestinal symptoms such as nausea, anorexia, weight loss, weakness, dizziness, and gas, together with mainly diarrhea or abdominal pain [7]. In addition, it was also reported that the parasite can be encountered together with serious cases such as rectal bleeding, weight loss, anemia, and eosinophilia [2]. For example, Carrascosa et al. reported a *B. hominis-*associated hemorrhagic proctosigmoiditis case [8]. *Blastocystis spp.*-induced superficial intestinal invasion and mucosal inflammation was demonstrated in animal studies [9] while the pathogenic role of *Blastocystis spp.* in bowel inflammation has been investigated in many clinical trials.

Although the association of *Blastocystis spp.* with irritable bowel syndrome (IBS) and inflammatory bowel disease (IBD) have been reported to be frequently encountered in daily practice, its role in the pathogenesis is still debated [10,11]. Therefore, the present study was designed to evaluate the prevalence of *Blastocystis spp.* in patients who applied to the same internal medicine-gastroenterology clinic with or without gastrointestinal complaints to reveal the association of this parasite with diagnosed IBS and IBD.

## Methods

### Study population

A total of 2526 patients who applied to the Gastroenterology Clinic of Antalya Training and Research Hospital from January 2010 to May 2011 were included in this retrospective case–control study based on presence of data on routine stool microscopy, stool culture and parasitic investigations by a gastroenterology specialist. Patients admitting with gastrointestinal complaints (n = 2334) including acute diarrhea, loss of appetite, dyspepsia, constipation, and abdominal pain composed the study group while a control group (n = 192) was selected from population attending the outpatient department within the similar time period on an outpatient basis for routine control without any accompanying gastrointestinal symptoms. Patients in the control group were not diagnosed with any gastrointestinal diseases (IBD, IBS etc.) and, they were also checked for fecal occult blood.

The patients who had positive stool culture results for pathogenic bacterias (*Campylobacter, Salmonella, Shigella, enteropathogenic E. coli* etc.) and the patients whose direct microscopic stool examination revealed pathogenic parasites (*Giardia lamblia, Entamoeba histolytica* etc.) were excluded from the study.

The study protocol was approved by the Ethics Committee of Antalya Training and Research Hospital.

### Assessments

IBS patients were diagnosed according to Rome III criteria the diagnosis of IBD was based on detailed clinical findings and endoscopic and histopathological examinations including ulcerative colitis (UC) and Crohn’s disease (CD). Stool specimens were evaluated microscopically after macroscopic analysis. For this purpose, native-Lugol and formol ethyl acetate concentration method was applied to these stool specimens initially, and suspicious cases were analyzed by trichrome staining method. Samples were prepared with native–Lugol, formol ethyl acetate concentration method, or trichrome staining method, and were examined under light microscope at X40, X10 or X100 magnification, respectively. All samples were evaluated by experienced parasitology specialists and the results were recorded. A sample was accepted as positive for *Blastocystis spp.* if five or more parasites were identified in every microscopic field at X40 magnification.

### Statistical analysis

Fisher’s exact test and Pearson chi-square analysis performed for categorical variables using the Statistical Package for the Social Sciences (SPSS) 13.0 and a two-sided p-value < 0.05 was considered statistically significant.

## Results

### Gastrointestinal diagnoses in the study group

Evaluation of the study group revealed the diagnosis of a gastrointestinal disease in 1212 patients including IBD (335, 14.4%) or IBS (877, 37.6%) based on clinical and laboratory tests. Of 335 patients with IBD, 276 patients were determined to have UC while CD disease was determined in 59 patients. A total of 32 patients with IBD were newly diagnosed during the study including 28 UC and 4 CD cases while rest of IBD patients (n = 303) had been followed as IBD previously.

### Frequency of Blastocystis spp. infections in the study vs. control group

Overall, *Blastocystis spp.* was found in 134 cases (5.74%) out of 2334 patients in the study group who applied to the gastroenterology clinic with gastrointestinal complaints while in 6 patients (3.12%) in the control group (n = 192) who applied to the same clinic without gastrointestinal complaints [Table 1]. There was no statistically significant difference between the study and control groups in terms of frequency of *Blastocystis spp.* (p = 0.128).

**Table 1:** **Demographic and clinical characteristics and ratio of*Blastocystis spp.*infections in study and control groups**

		**Symptomatic Patients**			**Control group**
	**IBS**	**IBD**	**Others**		
**Demographic and clinical characteristics**				
**Age (years); mean (SD)**	45.8(16.2)	45.2(13.2)	47.3(16.1)	45.5(15.2)
**Gender**				
Male%	39.6	41.8	44.9	42.4
Female%	60.4	58.2	55.1	57.6
**Clinical Symptoms%**				
Abdominal pain	79.8	83.5	81.1	
Constipation	50.2	14.9	49.5	
Diarrhea	34.2	89.5	48.8	
Bloating	79.8	74.6	71.3	
Flatulence	50.2	59.7	54.3	
***Blastocystis spp.*infections**				
	***B. hominis*positivity**			**Total**
	**N**	**%**		**N**
**Total symptomatic patients**	134	5.74		2334
Symptomatic patients/ undiagnosed	55	4.90		1122
IBD	28	8.35		335
UC	24	8.70		276
CD	4	6.78		59
IBS	51	5.82		877
**Controls**	6	3.12		192

IBS: Irritable bowel syndrome; IBD: Inflammatory bowel disease; UC: Ulcerative colitis; CD: Crohn’s disease.

Consideration of *Blastocystis spp.*, frequency in the study group with respect to diagnosis revealed the identification of *Blastocystis spp.* in significantly higher number of patients with IBD (28 of 335, 8.35%) patients when compared to control group (OR = 2.824; 95% CI: 1.149–6.944; p = 0.019). However there was no significant difference between patients with IBS (51 of 877 patients, 5.82%) and control group in terms of *Blastocystis spp.* frequency (p = 0.133). When the two types of IBD were evaluated in terms of *Blastocystis spp.* frequency compared to control group, *Blastocystis spp.* frequency was determined to be significantly higher in UC patients (24 of 276 UC patients, 8.7%) than control patients (OR = 2.952; 95% CI: 1.183-7.367; p = 0.016), while there was no significant difference between patients with CD (4 out of the 59 patients, 6.78%) and control group in terms of *Blastocystis spp.* frequency (p = 0.251). Only one UC patient - out of 32 newly diagnosed IBD patients - had *Blastocystis spp.* in their stool examinations. Considering patients admitting with gastrointestinal symptoms but not diagnosed with IBD or IBS (n = 1122), *Blastocystis spp.* was found in 4.9% (n = 55) of these patients and there was no statistical difference when compared with either the control group (p = 0.280) or the other symptomatic patients (p = 0.055) (Table 1).

### Demographic and clinical characteristics of patients and control group

The mean (SD) age of patients with IBS, patient with IBD, patients with gastrointestinal complaints and the control group were 45.8 (16.2), 45.2 (13.2), 47.3 (16.1), and 45.5 (15.2) years, respectively with no statistically significant difference between groups (p = 0.597). The groups were also comparable to each other in terms of gender (Table 1).

In patients with gastrointestinal complaints, gender distribution was homogenous in patients with (n = 134; 83 (62%) females and 51 (38%) males) or without *Blastocystis spp.* (n = 2200; 1260 (57.3%) females and 940 (42.7%) males) (p = 0.366).

## Discussion

*Blastocystis spp.* is the most common parasite found in human stool specimens [12] with prevalence reported to range from 1.5% and 10% in developed countries and from 30% and 50% in developing countries [1,2,13]. The parasite was encountered 28.5 times more than *Giardia lamblia* in a study conducted in the United States in 2000 [14]. In another study on the epidemiology of *Blastocystis spp.* conducted in the United States in 2006, 16,374 stool specimens were analyzed, and prevalence of *Blastocystis spp.* was found at a ratio of 18% [15]. In our study, the *Blastocystis spp.* ratios in patients with intestinal complaints and in the control group were 5.74% and 3.12%, respectively. This ratio is close to the ratio reported in the developed countries and also is in parallel with ratios (ranges from 1.4% to 44.3%) reported from Turkey to date. The fact that the criterion for *Blastocystis spp.* positivity has been accepted as the presence of five or more parasites in the microscopic field at X40 magnification in some of the studies, whereas consideration of positivity regardless of the number in others may explain the wide range of prevalence reported in the literature.

Although the specific pathogenicity of *Blastocystis spp.* is still undetermined, the intestinal inflammation induced by this parasite was reported to yield specific inflammatory changes in the gut wall. In this regard, in an experimental animal model study in mice, vacuolar form of the parasite was reported to cause invasion to the lamina propria, submucosal and muscular layers of the large intestine leading mixed inflammatory cell infiltration and active colitis in infected mice [16]. Although there are case reports on the pathogenicity of *Blastocystis spp.* in humans, the number of those studies is inadequate [17,18]. Nonetheless, proteases of *Blastocystis spp.* were considered a virulence factor that is responsible for protein degredation and have possible pathogenic role in host immune evasion [19].

Clinical manifestation of Blastocystis infection was reported to include asymptomatic, acute symptomatic and chronic symptomatic cases in the past studies based on use of different methologies and study populations. In a study in 2010, *B. hominis* was reported to be more frequent in patients with intestinal symptoms than in the asymptomatic patients (5.67% vs. 3.43%, respectively) [20]. In our study, albeit not statistically significant, frequency of blastocystis infection was also higher in symptomatic than asymptomatic patients (5.74% vs 3.12%). Researchers report that *Blastocystis spp.* is the most common parasite encountered in symptomatic patients based on two main reasons. Firstly, clinicians are reluctant to treat this parasite due to its low pathogenicity and self-restricting symptomatology; and secondly, *Blastocystis spp.* that are resistant to other antiparasitic medications have the ability to colonize easily into empty intestinal niches after the treatment of other pathogenic protozoa with conventional medications [7,21].

Although no clear correlation between diarrhea and *Blastocystis spp.* could be established by means of the controlled studies conducted to date, its relation with chronic bowel diseases of unknown origin such as IBD and IBS is currently being investigated due to the increasing prevalence of this parasite in developed countries.

Being a functional bowel disorder associated with abdominal pain or discomfort and defecation disorders, IBS is generally considered a psychosomatic disease, etiology of which has not been clarified yet [22]. IBS prevalence in developing countries varies between 35%-43% and it is a frequent disease in the clinical practice with 12% of the general medical visits and 25-50% of gastroenterology consultations resulting with the positive diagnosis [23]. Although IBS is a functional bowel disease, studies on the association between *Blastocystis spp.* and IBS revealed that parasite infection was evident in almost half of the patients with IBS [11,24,25]. Accordingly, Yakoob et al. reported that 52% of IBS patients and 24% of the control group had *Blastocystis spp.* infection [10]. In other related studies, *B. hominis* was found in 17.3% of symptomatic patients with IBS and in 6% of cases in the asymptomatic control group, and the difference between the two groups was reported to be significant [26]. In our study, patients with blastocystis infection and IBS demonstrated similar symptomatology in parallel with the literature. However, while there was a significant parallelism between the frequency of blastocystis infection and IBS prevalence in studies conducted in different countries, [24]. *Blastocystis spp.* was found in 51 (5.82%) of 877 symptomatic patients in our study with no statistically significant difference compared with the ratio obtained in the asymptomatic control group (3.12%) (p = 0.133).

The IBD sub-group (n = 335) of patients with gastrointestinal complaints (n = 2334) was determined to have the most frequent *Blastocystis spp.* infection (8.35%) with significantly higher rates compared with the controls (3.12%; p = 0.019) Likewise, 24 of 28 patients with *Blastocystis spp* in the IBD group were UC patients (n = 276) with significantly higher rate of infection (8.7%) in these patients compared to controls (3.12%, p = 0.016). However, while showing a tendency towards higher ratio in patients with CD, there was no statistical difference between CD patients (6.78%) and control group in terms of frequency of *Blastocystis spp.* infection (p = 0.251).

UC is a chronic inflammatory disease of the intestines manifesting itself with consequent activity and remission periods. Many studies have investigated the influence of intestinal infections on the clinical course of UC. In this respect, in a prospective case-controlled study by Yamamoto-Furusho et al. conducted in Mexico on the correlation of UC disease with parasite infections, *Blastocystis spp.* was reported as the most common parasite, at a rate of 10%, which is very similar to prevalence of parasite in our study (8.7%). They also reported that presence of *Blastocystis spp*., *Entamoeba histolytica* and *Entamoeba coli* was correlated with relapsing illness [27]. In another study, six UC patients with refractory symptoms were found to have *B. hominis* in their stool examinations with complete recovery by 14-day metronidazole treatment [28]. In a study by Mylonaki et al. emphasizing the importance of routine microscopic stool analysis in IBD exacerbation, *Blastocystis spp.* was amongst the detected enteric infectious agents [29]. These results suggested the correlation of *Blastocystis spp*. with IBD and strengthened the hypothesis that the parasite may have a role in the exacerbation of diseases [28]. In contrast, Nagler et al. reported that *Blastocystis spp.* was not a significant pathogen in IBD exacerbations. However, they examined only 12 patients retrospectively, so the lack of *Blastocystis spp.* might be associated with the inadequate number of patients examined [30].

In our study, a significant rate of *Blastocystis spp.* infection was detected in patients with gastrointestinal symptoms via common laboratory methods. However, there are studies reporting that the sensitivity of the standard methods used in clinical practice are lower than that of molecular methods and culture [31]. While it has long been accepted that *B. hominis* is the single blastocyst specie infecting humans, genetic analysis conducted recently demonstrated that nine different types of blastocyst may infect humans. Besides, antigenic heterogeneity of *Blastocystis spp.* supports the likelihood of virulent and avirulent species of this parasite [32]. In this regard, starting from the hypothesis of Boorom, more virulent and easily transmissible *Blastocystis spp.* may be the cause of the increase in the prevalence of IBDs in the 1990s in Europe [33] which indicates use of more sensitive methods such as polymerase chain reaction and culture in detection of more virulent types of this parasite.

The major limitation of the present study is the case–control design increasing the likelihood of confounding variables and bias (sampling and observation and recall bias). Nevertheless, a convenience sample (sampled in the same way as the cases) was selected from population attending the same outpatient department to overcome sampling bias and to enable the controls to represent the same population as the cases. Besides, the likelihood of retrospective recall bias seems to be minimized by using data recorded, for other purposes, before the outcome had occurred and therefore before the study had started. We didn’t assess the potential viral causes of the gastrointestinal complaints and this is the limitation of our study. Another limitation important in terms of clinical relevance of our findings is the neglect of including ulcerative colitis activation criteria in the diagnostic evaluation of patients in the study group. For this reason, albeit its clinical importance, we are unable to draw a conclusion regarding the causative role of Blastocystosis infection among UC patients admitting with exacerbation of the disease.

## Conclusions

*Blastocystis spp.* infection was more frequent in patients with IBD, especially those with UC. Although symptomatic IBS and CD patients had higher rates of *Blastocystis spp.* infection, there were no statistically significant differences when compared to the control group. We think that controlled studies on symptomatic patients and especially in UC patients will disclose the role of *Blastocystis spp.* in the pathogenesis as well as clinical course of IBS and IBD.

## Abbreviations

IBS: Irritable bowel syndrome; IBD: Inflammatory bowel disease; CD: Crohn’s disease; UC: Ulcerative colitis.

## Competing interests

The authors declare they have no conflict of interest.

## Authors’ contributions

AHC and YC designed the experiment, AHC participated in the selection of the study group patients, YA and ET participated in the selection of control group, FGK and YC were involved in the laboratory assessments, AHC, BOY and YC participated in the devolepment of the study. AHC and YC wrote the manuscript. BOY performed the statistical analysis. All authors read and approved the final manuscript.

## Pre-publication history

The pre-publication history for this paper can be accessed here:

http://www.biomedcentral.com/1471-230X/12/122/prepub
